# Anthracosilicosis Mimicking Primary or Secondary Lung Malignancy in a Patient With a History of Treated Prostate Cancer

**DOI:** 10.7759/cureus.99469

**Published:** 2025-12-17

**Authors:** Youseff Elrayes, Márton Mikó, Mohamed Hany Elrayes

**Affiliations:** 1 Radiology Department, Szabolcs-Szatmár-Bereg County Teaching Hospital, Nyíregyháza, HUN; 2 Radiology Department, East Kent Hospitals University NHS Foundation Trust, Canterbury, GBR

**Keywords:** anthracosilicosis, computed tomography, fdg-pet/ct, histopathology correlation, lung lesion, occupational lung disease, prostate cancer history, radiology-pathology correlation, silicosis, video-assisted thoracoscopic surgery biopsy

## Abstract

A 65-year-old male with a history of prostate adenocarcinoma treated with radical prostatectomy in 2013 was referred for evaluation of a new pulmonary lesion. Surveillance chest computed tomography (CT) revealed a newly developed 25 × 20 mm irregular parahilar mass in the right upper lobe. 18F-fluorodeoxyglucose positron emission tomography/computed tomography (FDG-PET/CT) showed intense uptake in the lesion (SUVmax 6.6) and mediastinal lymph nodes (SUVmax 12.4), raising concern for malignancy or metastatic recurrence. However, multiple bronchoscopic and mediastinoscopic biopsies were negative for malignancy. The patient subsequently underwent video-assisted thoracoscopic surgery (VATS) with partial pleural and parenchymal resection for definitive diagnosis. Histopathology showed fibrotic pleura, anthracotic pigment deposition, silica crystals under polarized light, and reactive sinus histiocytosis, confirming anthracosilicosis. No carcinoma was detected on pan-cytokeratin staining. A small right-sided pleural effusion persisted postoperatively but regressed gradually on follow-up imaging. This case highlights a diagnostic pitfall where FDG-PET/CT mimicked malignancy in a benign fibrosilicotic process, emphasizing the limitations of imaging alone and the critical importance of histopathologic confirmation before major surgical intervention.

## Introduction

The differentiation between malignant and benign pulmonary lesions remains one of the most challenging aspects of thoracic imaging. 18F-fluorodeoxyglucose positron emission tomography/computed tomography (FDG-PET/CT) is widely used for oncologic surveillance; however, its specificity is limited in the presence of granulomatous or fibrotic diseases, where inflammatory macrophage activity can produce high FDG uptake mimicking malignancy [1-5].

Occupational lung diseases such as silicosis and anthracosilicosis are notable mimickers of cancer due to their fibrotic nature, nodular parenchymal changes, and avid FDG uptake. In patients with a prior oncologic history, such findings can easily trigger invasive diagnostic or surgical procedures. We report a case of anthracosilicosis with postinflammatory fibrosis mimicking lung cancer in a patient previously treated for prostate carcinoma, highlighting the importance of multidisciplinary correlation and tissue confirmation.

## Case presentation

A 65-year-old male, a former bricklayer with prolonged occupational exposure to silica and dust, was under regular follow-up after radical prostatectomy for adenocarcinoma (2013). Prostate-specific antigen (PSA) levels remained persistently below detectable limits (<0.1 ng/mL) over a decade, indicating sustained remission.

**Figure 1:**
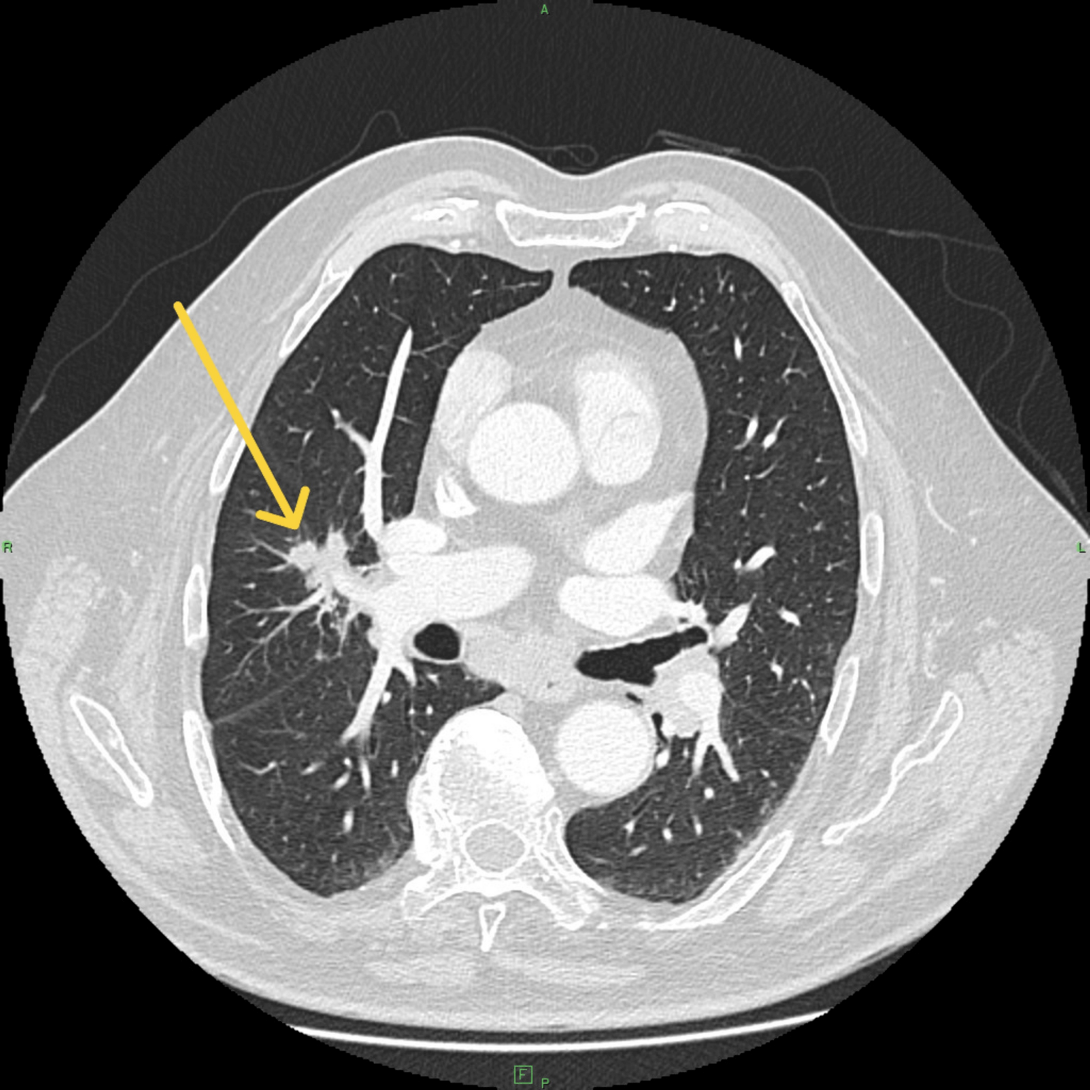
Axial contrast-enhanced CT image demonstrating a 25 × 20 mm irregular parahilar mass in the right upper lobe with spiculated margins, initially suspicious for malignancy.

**Figure 2:**
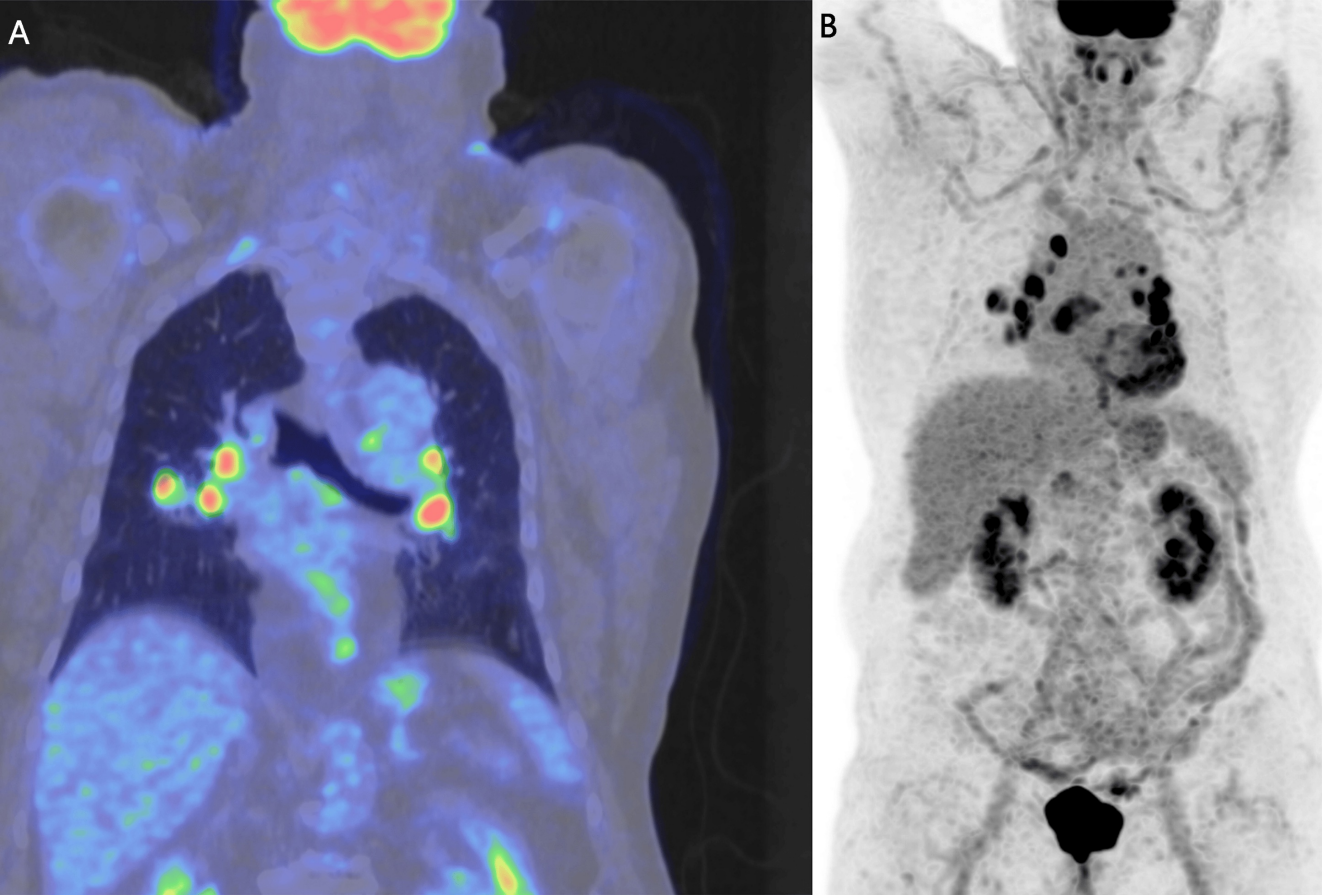
Coronal fused PET/CT (A) and whole-body PET maximum intensity projection (B). (A) showing intense FDG uptake in the right upper lobe parahilar lesion and mediastinal lymph nodes, with SUVmax values of approximately 6.6 and 12.4, respectively. The distribution of FDG-avid lymph nodes initially raised concern for metastatic disease. However, this pattern can also be seen in inflammatory conditions. Although malignancy could not be ruled out based on FDG uptake alone, subsequent histopathological analysis confirmed anthracosilicosis rather than neoplastic disease.

**Figure 3:**
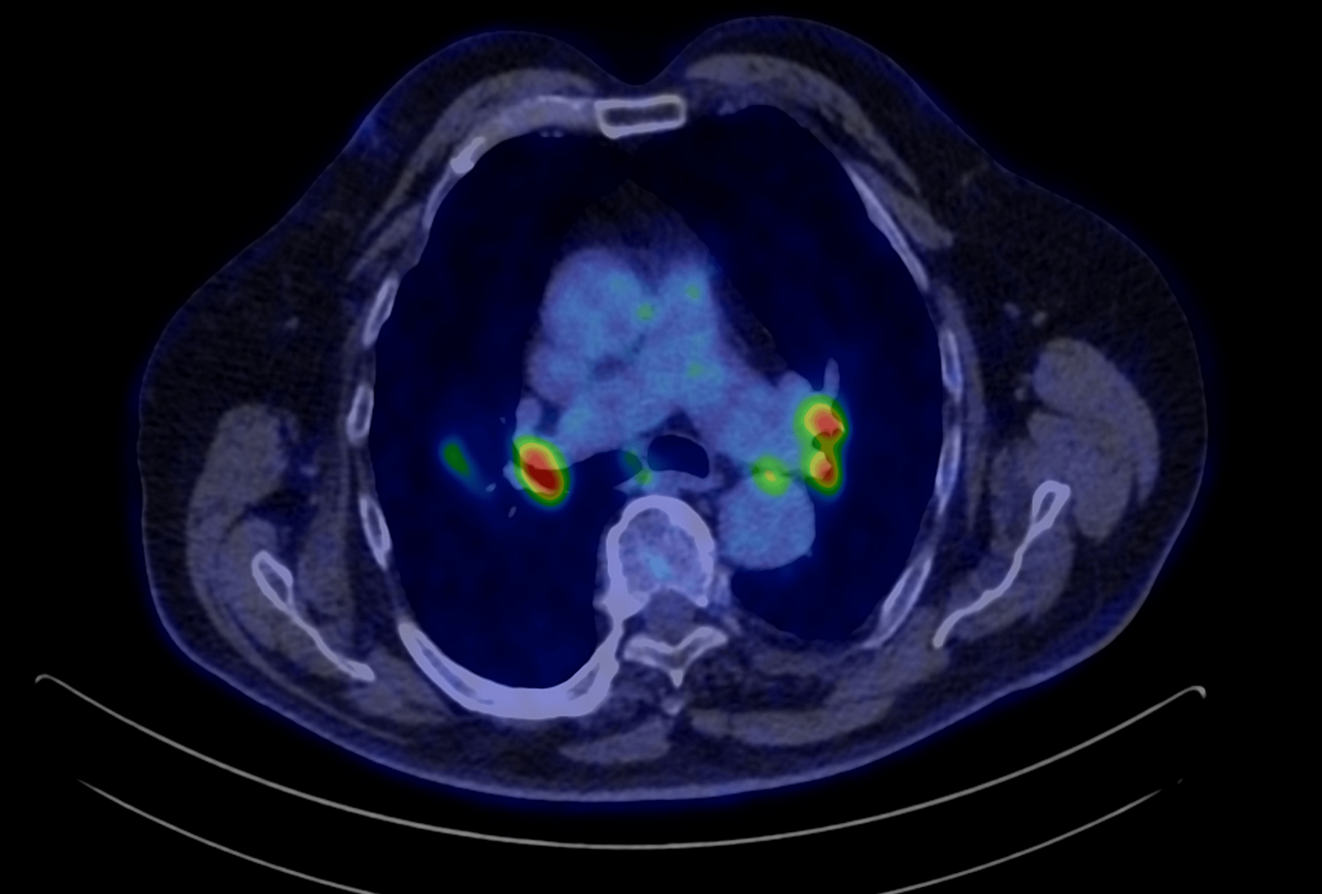
Axial 18F-fluorodeoxyglucose positron emission tomography/computed tomography (FDG-PET/CT) image. Axial-fused PET/CT image demonstrating bilateral mediastinal hypermetabolic lymph nodes with intense FDG uptake (SUVmax up to 12.4).

In late 2023, chest CT demonstrated a newly developed 25 × 20 mm irregular parahilar lesion in the right upper lobe (Figure 1). FDG-PET/CT revealed a hypermetabolic mass (SUVmax 6.6) with intensely active mediastinal lymph nodes (SUVmax 12.4) (Figures 2, 3). No abnormal FDG uptake was seen in the abdomen, pelvis, or skeleton. Laboratory results, including serum calcium, alkaline phosphatase, and bilirubin, were within reference ranges; C-reactive protein was mildly elevated at 9.2 mg/L.

**Figure 4:**
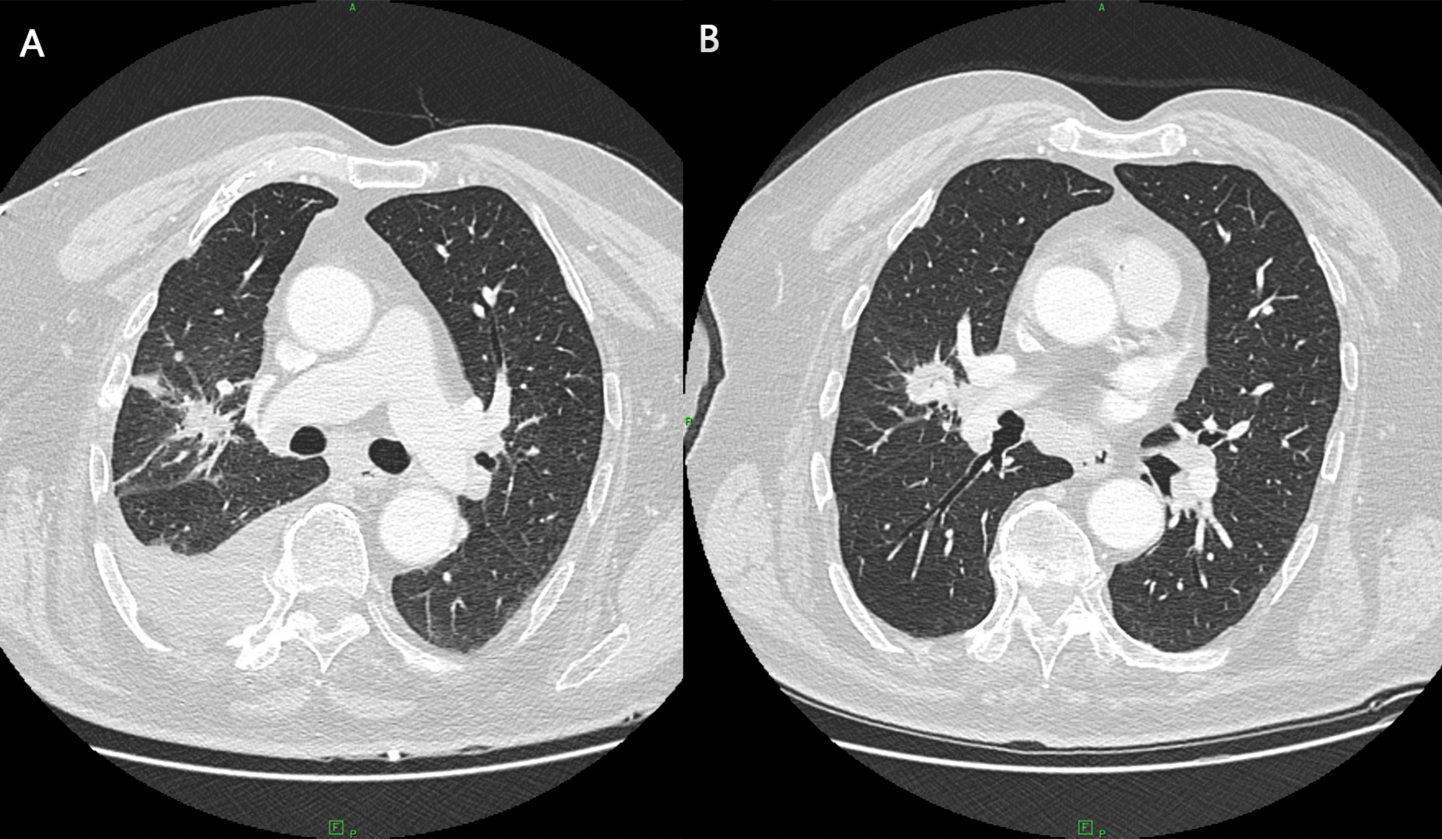
Postoperative (A) and preoperative (B) axial CT scans showing right parahilar lesion with irregular margins and plural fluid post VATS. (A) The parahilar lesion demonstrated irregular, fibrotic-appearing margins and (B) persisted without significant regression after video-assisted thoracoscopic surgery (VATS). (B) A mild right-sided pleural effusion was also noted postoperatively, gradually decreasing on subsequent follow-up imaging.

Bronchoscopy with exfoliative and brush cytology was negative for malignant cells, demonstrating only chronic bronchitis-related changes. Mediastinoscopy in February 2024 retrieved anthracotic lymph nodes with sinus histiocytosis but no carcinoma. Given the radiologic suspicion and persistent metabolic activity, diagnostic video-assisted thoracoscopic surgery (VATS) was performed in April 2024 with limited wedge resection of pleural and parenchymal tissue. A subsequent CT was performed, which showed an increase in the parahilar mass (Figure 4).

**Figure 5:**
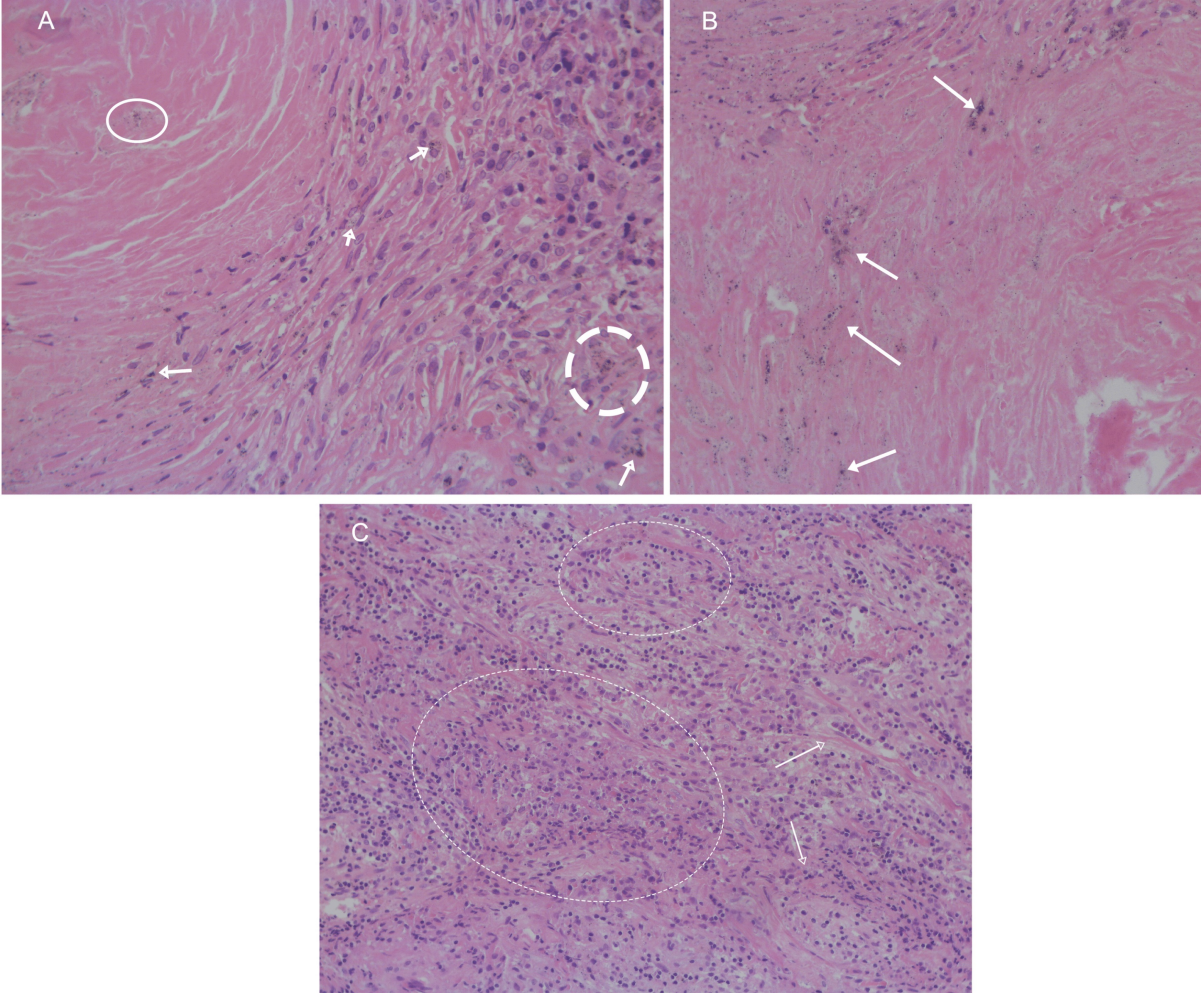
Lymph node biopsy (A) and anthra-silicotic nodules (B). Lung biopsy (C) showing chronic inflammatory infiltrates and foci of suppurative granulomatous inflammation.

**Figure 6:**
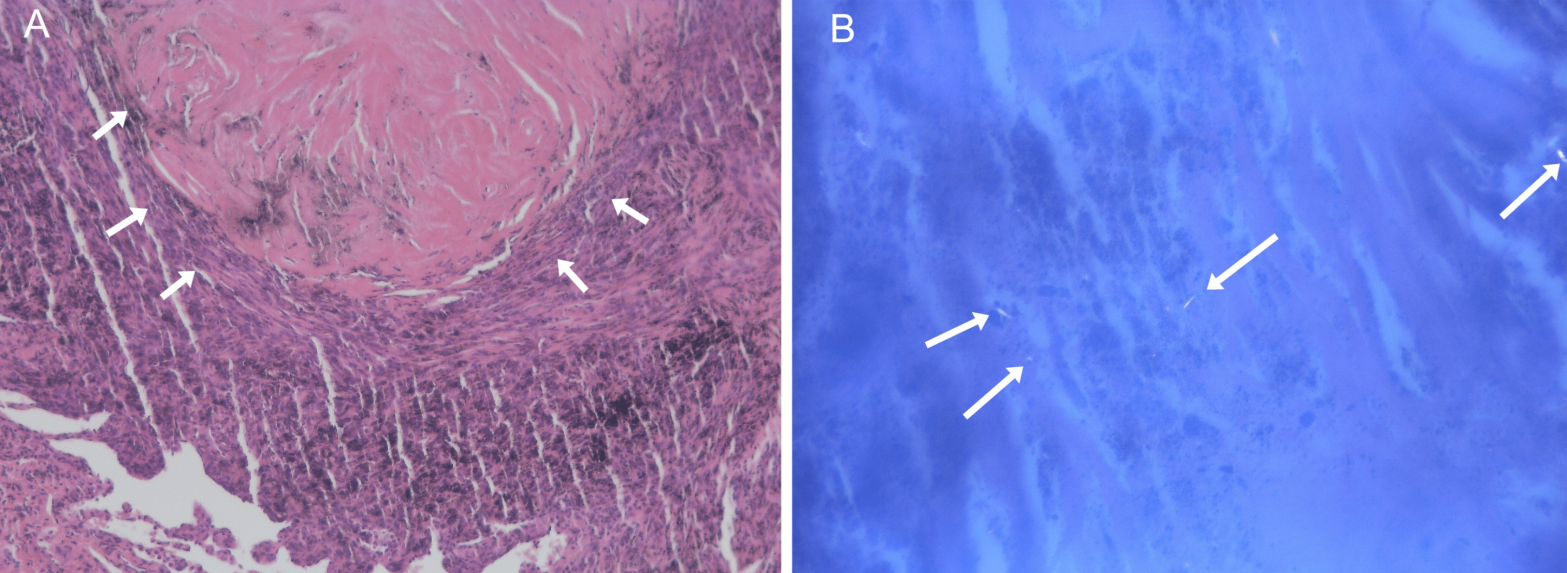
(A) Lung biposy showing typical anthro-silicotic nodule. (B) Silica crystals under polarized light section. Section of lung parenchyma showing a well-demarcated anthraco-silicotic nodule with dense collagenous fibrosis and abundant anthracotic pigment deposition (A). Polarized light microscopy highlights birefringent silica particles within the nodule (B).

Histopathological evaluation revealed anthracotic pigment, dense fibrotic nodules, and birefringent silica crystals under polarized light, consistent with anthracosilicosis. Pan-cytokeratin (pan-CK) immunostaining excluded epithelial malignancy. Chronic inflammatory infiltrates and small suppurative granulomas were observed, suggesting a reactive or low-grade infective component.(Figures 5, 6). No acid-fast bacilli were detected. Routine laboratory investigations were unremarkable. Serum PSA remained stable and undetectable (<0.1 ng/mL), effectively excluding prostate cancer recurrence. Liver and renal function tests were within normal limits. Mildly elevated inflammatory markers (CRP ≈ 9 mg/L) and slightly increased monocyte percentage (≈ 9%) were observed, but no significant hematologic or metabolic abnormalities were present (Table 1).

**Table 1:** Laboratory results with corresponding reference ranges The patient’s laboratory findings demonstrated a mildly elevated C-reactive protein (CRP) and slight monocytosis, while all other parameters, including prostate-specific antigen (PSA), calcium, alkaline phosphatase, and bilirubin, were within normal limits.

Test	Patient value	Units	Reference range	Comment
C-reactive protein (CRP)	9.2	mg/L	<5.0	Mildly elevated
Prostate-specific antigen (PSA)	< 0.1	ng/mL	0.0–4.0	Undetectable, excludes recurrence
Serum calcium	2.35	mmol/L	2.20–2.60	Normal
Alkaline phosphatase (ALP)	86	U/L	40–129	Normal
Total bilirubin	13	µmol/L	5–21	Normal
Monocytes (differential)	9	%	2–8	Slightly increased

**Figure 7:**
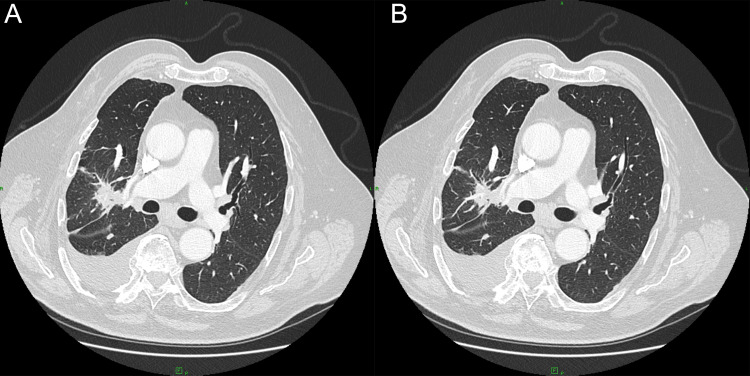
Follow-up-recent CT demonstrated residual parahilar densities without new nodules or interval progression. (A) taken in mid 2025 and (B) taken in late 2025.

Postoperatively, a small right-sided pleural effusion developed and gradually regressed on serial CT examinations (March and September 2025). Follow-up CT (Figure 7) in late 2025 showed a residual parahilar fibrotic band (23 × 19 mm, previously 29 × 22 mm) without new nodules or progression. The mediastinal nodes remained stable and subcentimetric. The patient remained clinically well, with stable respiratory function and no evidence of malignancy or metastasis.

## Discussion

This case underscores a critical diagnostic challenge: differentiating between FDG-avid benign pneumoconiosis and malignant pulmonary disease. FDG uptake reflects glucose metabolism and is not cancer-specific; activated macrophages in fibrosilicotic tissue can exhibit similar or even higher SUVs than certain neoplasms [1-3,6-9].

Several studies have reported false-positive PET/CT findings in anthracosis and silicosis, especially in patients with prior or concurrent malignancy [3,5,7]. The coexistence of mediastinal hypermetabolic nodes further compounds diagnostic uncertainty. In this patient, a prior oncologic history (prostate cancer) and the lesion’s morphological irregularity strongly favored malignancy, leading to invasive staging and ultimately surgical biopsy.

Histopathology confirmed anthracosilicosis, characterized by fibrotic nodules with silica crystals under polarized light and sinus histiocytosis. The presence of Langerhans histiocytes and suppurative granulomas indicated a mixed inflammatory-fibrotic process. These findings highlight the importance of occupational exposure history, which, when combined with PET findings, can mislead toward cancer in the absence of tissue proof [6-9].

Persistent mild pleural effusion following VATS is not uncommon in fibrosing pleuropulmonary disease and should not be misinterpreted as malignant recurrence. The absence of atypical cells, stable imaging over time, and consistent clinical stability further confirmed the benign nature of this process.

Serologic markers, although not markedly abnormal here, can occasionally complicate interpretation. Reports have described elevated neuron-specific enolase (NSE) or CA-125 levels in patients with silicosis or anthracosilicosis, even in the absence of malignancy [10-13]. Such biomarker elevations are attributed to macrophage activation and epithelial regeneration, mirroring the intensity of fibrotic inflammation rather than cancer progression.

This case aligns with prior publications emphasizing the overlap between inflammatory FDG uptake and malignant metabolism, reinforcing the principle that histopathologic confirmation remains the gold standard before committing to extensive resection or oncologic therapy [14-17].

## Conclusions

Anthracosilicosis can closely mimic pulmonary malignancy both morphologically and metabolically. In patients with prior cancer history, a multidisciplinary approach integrating radiologic, pathologic, and occupational data is vital to avoid unnecessary surgery. FDG-PET/CT, although sensitive, is not specific for malignancy and should be interpreted with caution in the presence of pneumoconiosis or chronic inflammation. This case illustrates that even intensely FDG-avid lesions may represent benign fibrosilicotic disease, and histopathology remains essential for definitive diagnosis.
